# Effects of gamma aminobutyric acid on performance, blood cell of broiler subjected to multi-stress environments

**DOI:** 10.5713/ab.22.0031

**Published:** 2022-09-02

**Authors:** Keun-tae Park, Mihyang Oh, Younghye Joo, Jong-Kwon Han

**Affiliations:** 1 Research and Development Center, Milae Bioresources Co., Ltd., Seoul 05836, Korea

**Keywords:** Broiler, Diurnal Temperature Range Stress, Gamma Aminobutyric Acid (GABA), Heat Stress, Overcrowding Stress

## Abstract

**Objective:**

Stress factors such as high temperatures, overcrowding, and diurnal temperature range exert profound negative effects on weight gain and productivity of broiler chickens. The potential of gamma aminobutyric acid (GABA) as an excitatory neurotransmitter was evaluated under various stress conditions in this study.

**Methods:**

The experiment was conducted under four different environmental conditions: normal, high temperature, overcrowded, and in an overcrowded-diurnal temperature range. The experimental groups were divided into (−) control group without stress, (+) control group with stress, and G50 group (GABA 50 mg/kg) with stress. Weight gain, feed intake, and feed conversion ratio were measured, and stress reduction was evaluated through hematologic analysis.

**Results:**

The effects of GABA on broilers in four experimental treatments were evaluated. GABA treated responded to environmental stress and improved productivity in all the experimental treatments. The magnitude of stress observed was highest at high temperature, followed by the overcrowded environment, and was least for the overcrowded-diurnal temperature range.

**Conclusion:**

Various stress factors in livestock rearing environment can reduce productivity and increase disease incidence and mortality rate. To address these challenges, GABA, an inhibitory neurotransmitter, was shown to reduce stress caused due to various environmental conditions and improve productivity.

## INTRODUCTION

Stress in the rearing environments of broiler significantly affects productivity. Examples of stress factors in general rearing environments of broiler include spatial limitations (rearing density), group rearing, environmental temperature, and light conditions. Prolonged stress can challenge the immunocompetence of animals, which can increase susceptibility to infectious diseases, and reduce growth, productivity, and the reproductive potential of broiler, all of which can significantly damage the economic feasibility of livestock production [1]. For example, 2014 had the highest temperature in recent times due to global warming and this heat led to a dramatic increase in broiler mortality worldwide, resulting in significant economic losses. High summer temperatures cause the most serious damage to broiler [2,3]. Once the external environmental temperature increases, broiler body temperature and respiratory rates also increase, leading to decreased feed intake and increased water consumption, which ultimately results in reductions of weight gain rate, reproduction rate, immunity, and an overall decline in productivity. In addition, the electrolyte balance in the blood is disrupted, which causes a hormone imbalance and poor nutritional state that leads to weight loss and productivity decline. More specifically, the laying ability of hens declines, egg weights decrease, and eggshells become thinner, causing an increase in eggshell breakage rate, and in this way, high temperatures are a detriment to the overall economy of the poultry industry [4].

Overcrowding and diurnal temperature range conditions are also important sources of continuous stress in broiler rearing, and have significant adverse effects on productivity and growth by elevating the risk of exposure to disease and temperature rise, respectively. Although insulation and ventilation equipment are required, the cost of these measures is significant and hence overcrowding and diurnal temperature range environments persist, prompting the need for improved regulatory measures. A primary target of stress stimulation is the hypothalamus, which secretes corticotropin-releasing hormone. This hormone acts on the adrenal cortex through the pituitary gland, and promotes the secretion of glucocorticoids, also known as the stress hormones. Increased glucocorticoid concentration in the blood is the main cause of homeostasis imbalance in animals.

Gamma-aminobutyric acid (GABA), a non-protein amino acid widely distributed in nature, is an excitatory-inhibitory neurotransmitter, which accounts for approximately 30% of neurotransmitters in the central nervous system [5]. GABA is found in the brain, kidneys, and heart of animals, and exists in grains, green teas, and rice embryos in plants. Significant GABA deficiency in the body results in excessive excitement and epileptic-like symptoms, and GABA is also the most important causative substance of depression [6,7]. In addition to neurotransmission, GABA has a variety of other physiological functions, including antihypertensive activity, suppression of insomnia, depression, and anxiety, memory enhancement, promotion of immunity against stress, and tranquilizing effects [8,9]. In addition, GABA plays a role in controlling the temperature of the hypothalamus. White rats that received a GABA injection were reported to have inhibited heat generation in the dorsomedial hypothalamus and posterior hypothalamus [10]. A large amount of GABA production from glutamate was reported, in which glutamate decarboxylase enzyme from the lactic acid bacterium Lactobacillus brevis was used [11], and studies to develop feed additives that can act as anti-stress reagents are now underway.

The purpose of this study was to investigate the potential use of GABA as an agent for tranquilization, digestion rate, and weight gain. In this study, GABA was provided alone as a functional feed additive to broiler chickens in a general environment, high temperature environment, overcrowding environment, and overcrowding-diurnal temperature range environment

## MATERIALS AND METHODS

### Animal care

All experimental procedures were approved by the Hankyung University Institutional Animal Care and Use Committee (Approval No.: 2015-02).

### Experimental reagent

The GABA (ML-F2) used in this experiment was produced in Milae Bioresources Co. Ltd. (Seoul, Korea), for which a *Lactobacillus* strain was fermented with glutamate as a substrate, and centrifuged after the fermentation, followed by spray drying of the supernatant. The components of the produced fermented product were 90% GABA, 8% yeast extract, 1% glucose, and 1% water.

### Experimental animals and experimental design

One-day old male broiler chicks (ROSS 308) were obtained from Yanji hatchery of rearing Co., Ltd. (Pyungtaek, Korea) and housed in iron wire cages in a broiler house immediately after delivery. The cages were equipped with a temperature control and nipple and the rearing area per replication was 540 cm^2^ (90 cm×60 cm). In total, 210 one-day-old (initial body) chicks were used for Trial 1, which were subjected to 3 treatments with 7 replications per treatment, and 10 chicks per replication by a completely random assignment. The temperature of the laboratory was set to 35°C on the first day, gradually decreased to 22°C by the 21st day, and maintained at 22°C from the 21st day to the 31st day when experiments terminated. Experimental groups were divided by treatment as follows: i) control group (CON, basal diet), ii) GB50 (GABA 50 mg/kg), iii) GB150 (GABA 150 mg/kg).

A high-temperature stress experiment was performed in Trial 2. In total, 360 one-day-old (initial body) male broiler chicks were subjected to 3 treatments with 12 replications per treatment, and 10 chicks per replication by a completely random distribution. Experimental groups were divided by treatment as follows: i) negative control group ((−) CON, basal diet), ii) positive control group ((+) CON, basal diet), and iii) GB50. The (−) CON was reared in a general environment without high temperature stress and the (+) CON and GB50 were reared in a high temperature environment from the 21st to 31st days. High temperatures were maintained at 33°C±2°C for 10 h, and then lowered to 22°C for 14 h. An electric water boiler was turned on at 08:00, and then the temperature was adjusted to 22°C at 08:00, 29°C at 10:00, 31°C at 12:00, 34°C at 14:00, 34°C at 16:00, and 33°C at 18:00. The boiler was turned off at 18:00, and then the temperature was set at 33°C at 18:00, 25°C at 19:00, and 22°C at 20:00 using a ventilator.

Trial 3 created an overcrowding environment (flock den sity, overcrowding stress) until the 31st day. In total, 480 one-day-old (initial body) male broiler chicks were subjected to 3 treatments with 12 replications per treatment by a completely random distribution. Experimental groups were divided by treatment as follows: i) (−) CON (10 chicks, 12 replications), ii) (+) CON (15 chicks, 12 replications), and iii) GB50 (15 chicks, 12 replications). The stress treatment was created by housing 150% of the recommended number of chicks in a single cage [12].

Trial 4 applied both overcrowding-diurnal temperature range stress simultaneously in the winter. The experimental method was the same as Trial 3, but the temperature difference between the day and the night was set to 20°C. Overcrowding-diurnal temperature range stress began on the 21st day when the early stage was terminated and extended to the 31st day, in which temperatures were kept at 30°C from 05:00 for 12 h, and then at 10°C from 17:00 for 12 h [13].

### Experimental feed and growth management

Chicks were provided with experimental feed *ad-libitum* and drank water freely during the experimental period. The feed used in the experiment was produced according to the growth standard of the Nutrient Research Composition (Table 1). Every diet was ground in order to mix the additives sufficiently and was provided as a powdered feed. For uniform mixing of GABA, the experimental feed was pulverized (size of about 600 μm) before mixing.

### Test items and methods

Experimental feed was provided until the 31st day, and then hematologic analysis was performed. Two chicks per cage were selected on average live weight, and 2 mL of blood was collected from the brachial vein with a 5 mL 23 gauge needle syringe. The collected bloods were transferred to vacuum blood tubes containing ethylene diamine tetraacetic acid (EDTA) and kept at 4°C until needed for hematologic analysis. Of the hematologic analysis items measured in Trials 2 through 4, hemoglobin (Hb), hematocrit (Hct), mean corpuscular volume (MCV), mean corpuscle hemoglobin (MCH), and mean corpuscular hemoglobin concentration (MCHC) were analysed using an XE-2100D Automated Hematology Analyzer (Sysmex, Japan) [14]. For white blood cell (WBC) and red blood cell counting, blood smears were made on a slide glass, and stained with Wright’s stain, followed by differential microscopic inspection under an optical microscope. For all growth experiments, the starter feed was provided until the 21st day, and the finisher feed was offered from the 22nd day to the 31st day. The weight of individual chicks was measured at the outset of the experiment, on the 21st day, and on the 31st day. Feed intake and feed conversion ratio were measured, recorded, and calculated every week.

### Statistical analysis

This experiments were conducted by completely randomized design. Analysis of variance was performed on all obtained results as a statistical analysis using the general linear model Program of Statistical Analysis System version 9.3 (SAS Institute, 2002). When a statistically significant difference was accepted by the analysis, the significance between treatments was tested at the significance level of p<0.05 using Duncan’s multiple range test.

## RESULTS

The effects of supplementing broiler chicken feed with GABA on weight gain, feed intake, and feed conversion ratio under general environment conditions are presented in Table 2 (Trial 1). There was no significant difference in productivity from the 1st day to the 21st day among treatment groups (p>0.05). In the GABA treatment groups, GB50 and GB150 improved the weight gain compared with the control group. However, GB50 improved approximately 2.3%, there was no significant difference on FCR. The GB50 treatment group had the highest feed intake during the entire period, and the feed conversion ratio showed an improvement despite the fact that there was no significant difference (p>0.05).

The results of supplementing feed with GB50 on weight gain, feed intake, and feed conversion ratio under a high temperature stress environment (Trial 2) are presented in Table 3. Weight gain was 664 g in the (−) CON and 660 g in the (+) CON during the early period, whereas GB50 gained the most at 688 g. The weight gains of the (−) CON, (+) CON, and GB50 group during the later (stress) period, were 584 g, 490 g, and 538 g, respectively. Hence, GB50 had a significant improvement in weight gain of approximately 9.8% compared to the (+) CON in the heat stress environment. Weight gain of the GB50 group was also in the same pattern during the entire period, in which the (+) CON showed a decrease by approximately 7.9% compared to the (−) CON, whereas the GB50 treatment group significantly decreased by approximately 1.8% compared to the (−) CON. There was no difference in feed intake among the groups during the early period, the (−) CON and GB50 group showed a significant increase in feed intake compared to the (+) CON in the later period, which had consistent feed intake throughout the experimental period. Feed conversion ratio of the (−) CON and GB50 group were 1.52 and 1.58, respectively, whereas it significantly increased to 1.64 in the (+) CON. Hematologic analysis results of GB50 supplementation under the high temperature stress environment are presented in Table 4. There were no differences in the values of red blood cells, MCV, MCH, and MCHC under the high temperature stress environment, whereas the Hct count significantly decreased in the (+) CON and GB50 group compared to the (−) CON. In addition, the WBC count increased by 269.6 in the (−) CON and 489.5 in the (+) CON, corresponding to approximately 81.5%, whereas the GB50 group showed a significant increase to 411.4 corresponding to approximately 52.6% compared to the (−) CON (p<0.05).

The effects of supplementing feed with GB50 on weight gain, feed intake, and feed conversion ratio under overcrowding stress conditions (Trial 3) are presented in Table 5. There was no change in weight gain during the early period, the weight of the (−) CON without stress was 596 g, and the weights of the (+) CON and GB50 group (with overcrowding stress) were 432 g and 488 g, respectively, during the later period, showing significant reductions of approximately 27.5% and 18.1%, respectively, compared to the (−) CON. The (+) CON and GB50 group also showed significant weight reductions of approximately 13.5% and 7.1% over the entire experimental period, respectively. There was no difference in feed intake among the groups during the early period, whereas feed intake significantly decreased in the (+) CON and GB50 treatment group during the later period compared to the (−) CON. Feed efficiencies during the later period were 1.55 in the (−) CON and 1.98 in the (+) CON, which was a dramatic increase of approximately 27.7%, whereas the efficiency of the GB50 treatment group was 1.78, corresponding to a 14.8% increase. GB50 supplementation improved feed efficiency under stressful conditions. Hematologic analysis results of supplementing feed with GB50 in an overcrowded stress environment are presented in Table 6. The WBC count in the (+) CON increased by approximately 49% compared to that of the (−) CON, whereas the WBC count of the GB50 treatment group increased by 22.7%, showing that GB50 treatment suppressed the elevation of WBC count caused by overcrowding stress. In addition, only the (+) CON showed a significant reduction in Hct (p<0.05).

The effects of supplementing feed with GB50 on weight gain, feed intake, and feed conversion ratio under overcrowding-diurnal temperature range stress environment are presented in Table 7 (Trial 4). Weight gain in the (−) CON, (+) CON, and GB50 treatment group during the early period were 642, 640, and 683 g, respectively, with only the GB50 group showing a significant increase of 6.4%. However, the (−) CON showed the highest weight gain in the later period, whereas the GB50 treatment group had a significant decrease, and the (+) CON had an even greater reduction. There was no difference in feed intake during the early period, the (−) CON had 948 g, the highest in the later period, and the (+) CON and GB50 treatment group had 736 and 778 g, respectively, which was a significant reduction compared to (−) CON. However, there was no significant difference among stress treatment groups.

Feed efficiency showed no difference in the early period. In contrast, feed efficiencies of the (+) CON and GB50 group (stress treatment groups), were 2.10 and 1.88 during the later period, which were significant increases compared to the (−) CON that had a feed efficiency of 1.53. However, the addition of GB50 significantly improved the feed conversion ratio compared to the (+) CON, which had a consistent feed efficiency throughout the entire experimental period. Hematologic analysis results of supplementing feed with GB50 under overcrowding-diurnal temperature range stress environment are presented in Table 8. The WBC count in the (+) CON increased by approximately 45.7% compared to the (−) CON, whereas it increased by 15.3% in the GB50 group, so that GB50 treatment suppressed the elevation of WBC associated with overcrowding-diurnal temperature range stresses. In addition, only the (+) CON showed a significant decrease in Hct (p<0.05).

## DISCUSSION

The present study investigated the effects of supplementing broiler chicken feed with GABA on productivity under a high temperature stress environment. High temperatures negatively affects poultry as well as other livestock, as evidenced by productivity decline, feed intake reduction, and death [15–17], and therefore this study evaluated how the supplementation of GABA in feed may reduce high temperature stress and improve productivity.

A characteristic of broiler chickens reared under high temperature stress is a rapid decline of weight gain after the early period. According to multiple reports on the rearing of broiler chickens and turkeys, body weight, feed intake, and feed conversion ratio decreased in high temperature environments compared to a general environment [18,19], and the present study also confirmed these phenomena. The present study identified the effects of the addition of a single supplement, GABA (GB50, GB150).

Stress in chickens has been reported to cause reductions in the level of GABA receptors in the brain [20], and this response within the pituitary gland reduces feed intake and body weight [21]. In addition, broiler chickens reared in a high temperature environment experience decreases in serum GABA levels and reduced weight gain. However, when GABA was added to the feed, body weight and GABA levels in the serum both greatly increased [22]. In the present study, when 50 mg/kg of GABA was supplemented in the feed under high temperature conditions, weight gain increased by approximately 4.2% in the early period and approximately 10.8% in the later period compared to the (+) CON, and when fed as a single agent, no effect on weight gain in the total period was complemented. Changes in blood characteristics attributed to high temperature stress include an elevated WBC count and reductions in Hct and Hb [23]. An elevation of body temperature by high temperature stress can greatly increase water intake causing blood dilution, which can reduce Hct and Hb counts [2,24]. The present study showed an approximately 81.6% increase in WBC count under high temperature conditions compared to the general environment, whereas the WBC count significantly increased by approximately 52.6% in the GB50 treatment group, confirming that blood indices are effective in the reduction of high temperature stress. In addition, the Hct count decreased significantly more under the high temperature environment than under the general environment, consistent with previous reports, whereas supplementation with GB50 did not elevate the Hct count. Moreover, whereas the Hb count was also significantly reduced by high temperature stress, GB50 supplementation did not increase the Hb count. The overcrowding stress experiment (Trial 3) found no significant differences in weight gain, feed intake, and feed efficiency during the early period. However, GB50 addition significantly improved weight gain and feed efficiency during the later period. In particular, whereas the (+) CON and GB50 treatment group had a similar feed intake during the entire experimental period, the GB50 treatment group showed an improvement in feed efficiency of approximately 10.1% owing to a 7.4% body weight increase. Although the WBC count showed a similar pattern to that of Trial 2, the elevation of the count was less than that of high temperature stress, and the WBC count was more sensitive to high temperature than overcrowding stress. In addition, the Hct count showed a significant decrease in the (+) CON compared to the (−) CON, but the GB50 treatment group showed no difference.

The cage area used in this experiment was 5,400 cm ^2^. The areas occupied by (+) CON and GB50 groups were 54 cm^2^ and 36 cm^2^, respectively. The area occupied by (+) CON group broiler chickens were approximately 33.3% lesser than that of the GB50 group. There were five nipples across the center of the cage ceiling. It is possible that some broilers occupied the nipples and fed more owing to their rank in the narrow cage. Hence, the intake of the remaining broilers may have decreased due to poor access to feed and water. Therefore, the reason for the weight loss in the GB50 group could be attributed to limited access to feed and drinking water.

It can be considered that GABA may have increased the ability of broiler to adapt to environmental stress. GABA is present in the enteric nervous peripheral nervous system through the gut-brain axis. GABA is a major inhibitory neurotransmitter in the mammalian brain. GABA is a multifunctional molecule that has different situational functions in the environment. In this study, GABA may have acted on the peripheral nerves present in the skin of broilers. Therefore, the pain, weight reduction, and physiological changes caused by various stresses were reduced.

The experiment with overcrowding stress in a diurnal temperature range environment (Trial 4) had results that were similar to those of Trial 3. However, the addition of diurnal temperature range stress remarkably decreased the weight gain of the (+) CON and GB50 treatment group compared to those of Trial 3, and feed conversion ratio also significantly decreased, which indicates that the diurnal temperature range stress challenge was successful.

The weight loss of the group exposed to the diurnal tem perature range was likely due to a decrease in feed intake due to extreme environmental changes [25]. In general, broiler feed intake decreases as the temperature environment decreases. They clustered to avoid heat loss and the frequency for access to the feed and water was reduced. This factor is also expected to affect the weight loss of the exposed group.

The present study evaluated 4 environmental variables, including general environment, high temperature stress environment, overcrowding stress environment, and overcrowding stress in diurnal temperature range environment, in which weight gain of the (+) CON during the entire period decreased by 7.9%, 13.5%, and 21.0%, respectively, compared to those of the (−) CON. However, the addition of GB50 increased weight gain by 6.6%, 7.4%, and 10.8%, respectively, compared to the (+) CON, indicating that GB50 was more effective in a more stressful environment. High temperature is a commonly regarded form of livestock stress, and hence it is considered the most serious challenge to these animals. However, the present study performed experiments under three stress environments, and although the severity of stress was not the same among the groups, the stress environment was the only variable among the groups. Therefore, it is impossible to perform an absolute comparison to determine the most severe stress factors. However, it could be assumed that the magnitude of stress increased in the order of high temperature < overcrowding < overcrowding-diurnal temperature range.

## CONCLUSION

In conclusion, various stresses in livestock rearing environments can reduce productivity and increase disease incidence and mortality rate. In order to address these phenomenon, GABA, an inhibitory neurotransmitter, was shown to prevent various stresses and improve productivity. However, this was a preliminary study on the development of anti-stress agents against multiple simultaneous stresses, and hence these results need to be confirmed by additional analyses on stress indices.

## Figures and Tables

**Table 1:** Composition of the basal diets for trials 1 to 4 (%)

Ingredients	Starter (1 to 21 d)	Finisher (22 to 42 d)
Corn, yellow	53.90	57.60
Wheat bran	5.00	5.00
Soybean meal (46% CP)	23.60	21.00
Full-fat soybean (extruded)	5.00	3.00
Fish meal (55%)	2.00	2.00
Poultry by products meal	2.00	3.00
Tallow	4.90	4.90
Salt	0.24	0.24
Dicalcium phosphate	1.00	1.00
Limestone	1.38	1.32
Vitamin, mineral premix^1)^	0.15	0.15
L-lysine-HCl (50%)	0.45	0.39
DL-methionine	0.31	0.25
L-threonine	0.07	0.05
Choline-chloride	0.05	0.05
Coccidiostat^2)^	0.05	0.05

1) The vitamin and mineral premix contained per kilogram of diet: vit A, 15,000 IU; vit D_3_, 7,500 IU; vit K_3_, 4.5 mg; vit B, 3 mg; vit B_2_, 9 mg; vit B_12_, 0.024 mg; niacin, 75 mg; Ca-pantothenate, 19.5 mg; Folic acid, 19.5 mg; Cu, 7.5 mg; I, 1.875 mg; Mn, 165 mg; Zn, 150 mg; Se, 0.45 mg, Fe, 60 mg; Co, 7.5 mg.

2) Maduramycin, Trade name: Cocstar, Daone chemical Co., Seoul, Korea.

**Table 2:** Growth performance, feed intake and feed conversion ratio of broiler chickens (Trial 1)

Items	Treatment^1)^			Pooled SEM

	CON	GB50	GB150	
Weight gain (g)				
1 to 21 d	681^a^	715^a^	712^a^	28.2
21 to 31 d	598^a^	612^a^	600^a^	42.3
1 to 31 d	1,279^b^	1,327^a^	1,312^a^	92.7
Feed intake (g/bird)				
1 to 21 d	990^a^	1,003^a^	1,011^a^	36.2
21 to 31 d	989^a^	1,008^a^	998^a^	74.8
1 to 31 d	1,979^a^	2,011^a^	2,009^a^	107.6
FCR (feed/gain)				
1 to 21 d	1.45^a^	1.40^a^	1.41^a^	0.11
21 to 31 d	1.65^a^	1.65^a^	1.66^a^	0.15
1 to 31 d	1.55^a^	1.52^a^	1.53^a^	0.18

SEM, standard error of the mean; FCR, feed conversion ratio.

1) (−) CON, control group without stress; (+) CON, control group with stress; GB50; gamma aminobutyric acid 50 mg/kg; GB150, gamma aminobutyric acid 150 mg/kg.

a,b Means within rows with no common superscript differ significantly (p<0.05).

**Table 3:** Growth performance, feed intake and feed conversion ratio of broiler chickens in heat stress environment (Trial 2)

Items	Treatment^1)^			Pooled SEM

	(−) CON	(+) CON	GB50	
Weight gain (g)				
1 to 21 d	664^ab^	660^b^	688^a^	92.6
21 to 31 d	584^a^	490^c^	538^b^	74.2
1 to 31 d	1,249^a^	1,150^c^	1,226^b^	166.8
Feed intake (g/bird)				
1 to 21 d	983^a^	983^a^	996^a^	136.3
21 to 31 d	927^a^	904^b^	944^a^	127.7
1 to 31 d	1,907^a^	1,887^b^	1,940^a^	263.9
FCR (feed/gain)				
1 to 21 d	1.48^a^	1.49^a^	1.45^b^	0.20
21 to 31 d	1.58^c^	1.84^a^	1.75^b^	0.23
1 to 31 d	1.52^b^	1.64^a^	1.58^b^	0.21

SEM, standard error of the mean; FCR, feed conversion ratio.

1) (−) CON, control group without stress; (+) CON, control group with stress; GB50; gamma aminobutyric acid 50 mg/kg.

a–c Means within rows with no common superscript differ significantly (p<0.05).

**Table 4:** Effects of heat stress on blood cell count (Trial 2)

Treatment^1)^	white blood cell (10^2^/μL)	red blood cell (10^6^/μL)	Hemoglobin (g/dL)	Hematocrit (%)	MCV (fL)	MCH (pg)	MCHC (g/dL)
(−) CON	296.9^c^	2.4	8.0^a^	35.3^a^	142.4	33.1	23.2
(+) CON	489.5^a^	2.4	7.6^b^	32.5^b^	142.0	32.6	22.9
GB50	411.4^b^	2.4	7.6^b^	32.6^b^	142.2	32.4	22.8
Pooled SEM	43.16	0.25	0.83	1.62	15.37	3.53	2.48

MCV, mean corpuscular volume; MCH, mean corpuscular hemoglobin; MCHC, mean corpuscular hemoglobin; SEM, standard error of the mean.

1) (−) CON, control group without stress; (+) CON, control group with stress; GB50; gamma aminobutyric acid 50 mg/kg.

a–c Means within columns with no common superscript differ significantly (p<0.05).

**Table 5:** Growth performance, feed intake and feed conversion ratio of broiler chickens in overcrowding environment (Trial 3)

Items	Treatment^1)^			Pooled SEM

	(−) CON	(+) CON	GB50	
Weight gain (g)				
1 to 21 d	677^a^	669^a^	694^a^	80.13
21 to 31 d	596^a^	432^c^	488^b^	59.54
1 to 31 d	1,273^a^	1,101^c^	1,182^b^	139.67
Feed intake (g/bird)				
1 to 21 d	988^a^	974^a^	986^a^	115.79
21 to 31 d	926^a^	856^b^	867^b^	104.05
1 to 31 d	1,914^a^	1,830^b^	1,853^b^	219.85
FCR (feed/gain)				
1 to 21 d	1.46^a^	1.46^a^	1.42^a^	0.17
21 to 31 d	1.55^c^	1.98^a^	1.78^b^	0.20
1 to 31 d	1.50^c^	1.66^a^	1.61^a^	0.18

SEM, standard error of the mean; FCR, feed conversion ratio.

1) (−) CON, control group without stress; (+) CON, control group with stress; GB50; gamma aminobutyric acid 50 mg/kg.

a–c Means within rows with no common superscript differ significantly (p<0.05).

**Table 6:** Effects of overcrowding stress on blood cell count (Trial 3)

Treatment^1)^	White blood cell (10^2^/μL)	Red blood cell (10^6^/μL)	Hemoglobin (g/dL)	Hematocrit (%)	MCV (fL)	MCH (pg)	MCHC (g/dL)
(−) CON	209.8^c^	2.4	8.5	30.4^a^	142.4	33.1	23.2
(+) CON	312.5^a^	2.3	8.4	29.1^b^	142.0	32.6	22.9
GB50	257.6^b^	2.5	8.6	31.4^a^	142.2	32.4	22.8
Pooled SEM	31.92	0.33	0.17	4.17	19.60	4.50	3.16

MCV, mean corpuscular volume; MCH, mean corpuscular hemoglobin; MCHC, mean corpuscular hemoglobin; SEM, standard error of the mean.

1) (−) CON, control group without stress; (+) CON, control group with stress; GB50; gamma aminobutyric acid 50 mg/kg.

a–c Means within columns with no common superscript differ significantly (p<0.05).

**Table 7:** Growth performance, feed intake and feed conversion ratio of broiler chickens in overcrowding and diurnal temperature range environment (Trial 4)

Items	Treatment^1)^			Pooled SEM

	(−) CON	(+) CON	GB50	
Weight gain (g)				
1 to 21 d	642^b^	640^b^	683^a^	77.18
21 to 31 d	612^a^	530^c^	414^b^	61.11
1 to 31 d	1,254^a^	990^c^	1,097^b^	131.23
Feed intake (g/bird)				
1 to 21 d	988^a^	948^a^	988^a^	114.85
21 to 31 d	936^a^	736^b^	778^b^	96.23
1 to 31 d	1,884^a^	1,684^c^	1,766^b^	209.51
FCR (feed/gain)				
1 to 21 d	1.48^a^	1.48^a^	1.45^a^	0.20
21 to 31 d	1.53^c^	2.10^a^	1.88^b^	0.25
1 to 31 d	1.50^c^	1.70^a^	1.61^b^	0.22

SEM, standard error of the mean; FCR, feed conversion ratio.

1) (−) CON, control group without stress; (+) CON, control group with stress; GB50; gamma aminobutyric acid 50 mg/kg.

a–c Means within rows with no common superscript differ significantly (p<0.05).

**Table 8:** Effects of overcrowding and diurnal temperature range stress on blood cell count (Trial 4)

Treatment	White blood cell (10^2^/μL)	Red blood cell (10^6^/μL)	Hemoglobin (g/dL)	Hematocrit (%)	MCV (fL)	MCH (pg)	MCHC (g/dL)
(−) CON	212.8^c^	2.4	8.3^a^	34.3^a^	127.7	32.8	25.9
(+) CON	308.9^a^	2.6	7.4^b^	32.4^b^	125.3	32.4	25.8
GB504)	244.4^b^	2.7	8.0^a^	34.2^a^	128.0	33.1	26.0
Pooled SEM	35.20	0.35	1.08	4.72	17.50	4.51	3.56

MCV, mean corpuscular volume; MCH, mean corpuscular hemoglobin; MCHC, mean corpuscular hemoglobin; SEM, standard error of the mean.

1) (−) CON, control group without stress; (+) CON, control group with stress; GB50; gamma aminobutyric acid 50 mg/kg.

a–c Means within columns with no common superscript differ significantly (p<0.05).
